# Income disparities in driving distance to health care infrastructure in the United States: a geographic information systems analysis

**DOI:** 10.1186/s13104-022-06117-w

**Published:** 2022-06-27

**Authors:** Jingchuan Guo, Inmaculada Hernandez, Sean Dickson, Shangbin Tang, Utibe R. Essien, Christina Mair, Lucas A. Berenbrok

**Affiliations:** 1 Department of Pharmaceutical Outcomes and Policy, University of Florida College of Pharmacy, 1225 Central Drive HPNP 2338, Gainesville, FL USA; 2 Division of Clinical Pharmacy, Skaggs School of Pharmacy and Pharmaceutical Sciences, University of California at San Diego, La Jolla, San Diego, CA USA; 3 West Health Policy Center, Washington, DC USA; 4 Division of General Internal Medicine, University of Pittsburgh School of Medicine, Pittsburgh, PA USA; 5 Department of Behavioral and Community Health Sciences, Graduate School of Public Health, University of Pittsburgh, Pittsburgh, PA USA; 6 Department of Pharmacy and Therapeutics, University of Pittsburgh School of Pharmacy, Pittsburgh, PA USA

**Keywords:** Health disparities, Low income, Health care access, Health care infrastructure

## Abstract

**Objective:**

Inequities in access to health care contribute to persisting disparities in health care outcomes. We constructed a geographic information systems analysis to test the association between income and access to the existing health care infrastructure in a nationally representative sample of US residents. Using income and household size data, we calculated the odds ratio of having a distance > 10 miles in nonmetropolitan counties or > 1 mile in metropolitan counties to the closest facility for low-income residents (i.e., < 200% Federal Poverty Level), compared to non-low-income residents.

**Results:**

We identified that in 954 counties (207 metropolitan counties and 747 nonmetropolitan counties) representing over 14% of the US population, low-income residents have poorer access to health care facilities. Our analyses demonstrate the high prevalence of structural disparities in health care access across the entire US, which contribute to the perpetuation of disparities in health care outcomes.

## Introduction

Inequities in access to health care contribute to persisting disparities in health care outcomes. This has been highlighted by the Coronavirus Disease 2019 (COVID-19) pandemic, which has disproportionately affected racial and ethnic minority groups and socially disadvantaged communities. Income disparities in access to health care and health outcomes have been widely documented over the decades [1–3]. Previous studies have also demonstrated that low spatial access to care was associated with poor health care and outcomes [4]. However, there is little evidence to address whether income disparities in spatial access to health exist across the US counties. We therefore constructed a geographic information systems analysis to test the association between income and access to the existing health care infrastructure in a nationally representative sample of US residents.

## Main text

### Materials and methods

We obtained addresses of rural health centers and hospital outpatient departments from the Centers for Medicare and Medicaid Services, of community pharmacies from the National Council for Prescription Drug Programs, and of federally qualified health centers from the Health Resources and Services Administration. The US population was characterized with the 2010 US Synthetic Population by RTI International.

We used ArcGIS Network Analyst and the US Geological Survey National Transportation Dataset [2] to estimate the driving distance to the closest facility for a 1% sample of the population, as previously described. [3] Using income and household size data, we calculated the odds ratio of having a distance > 10 miles in nonmetropolitan counties or > 1 mile in metropolitan counties to the closest facility for low-income residents, compared to non-low-income residents. Low income was defined as household income < 200% Federal Poverty Level [4]. The 1 mile cutoff in metropolitan counties and 10 miles cutoff in nonmetropolitan counties were selected based on definitions of food deserts by US Department of Agriculture [5]. Metropolitan and nonmetropolitan counties were classified following the National Center for Health Statistics Urban–Rural Classification Scheme for Counties [5].

## Results

Among 2,982,544 residents in the national sample, 32.4% were categorized as low-income and 14.8% lived in nonmetropolitan counties. Approximately 75% of the nonmetropolitan county residents lived within 10 miles of a facility, and 53% of the metropolitan county residents lived within 1 mile of a facility.

**Fig. 1:**
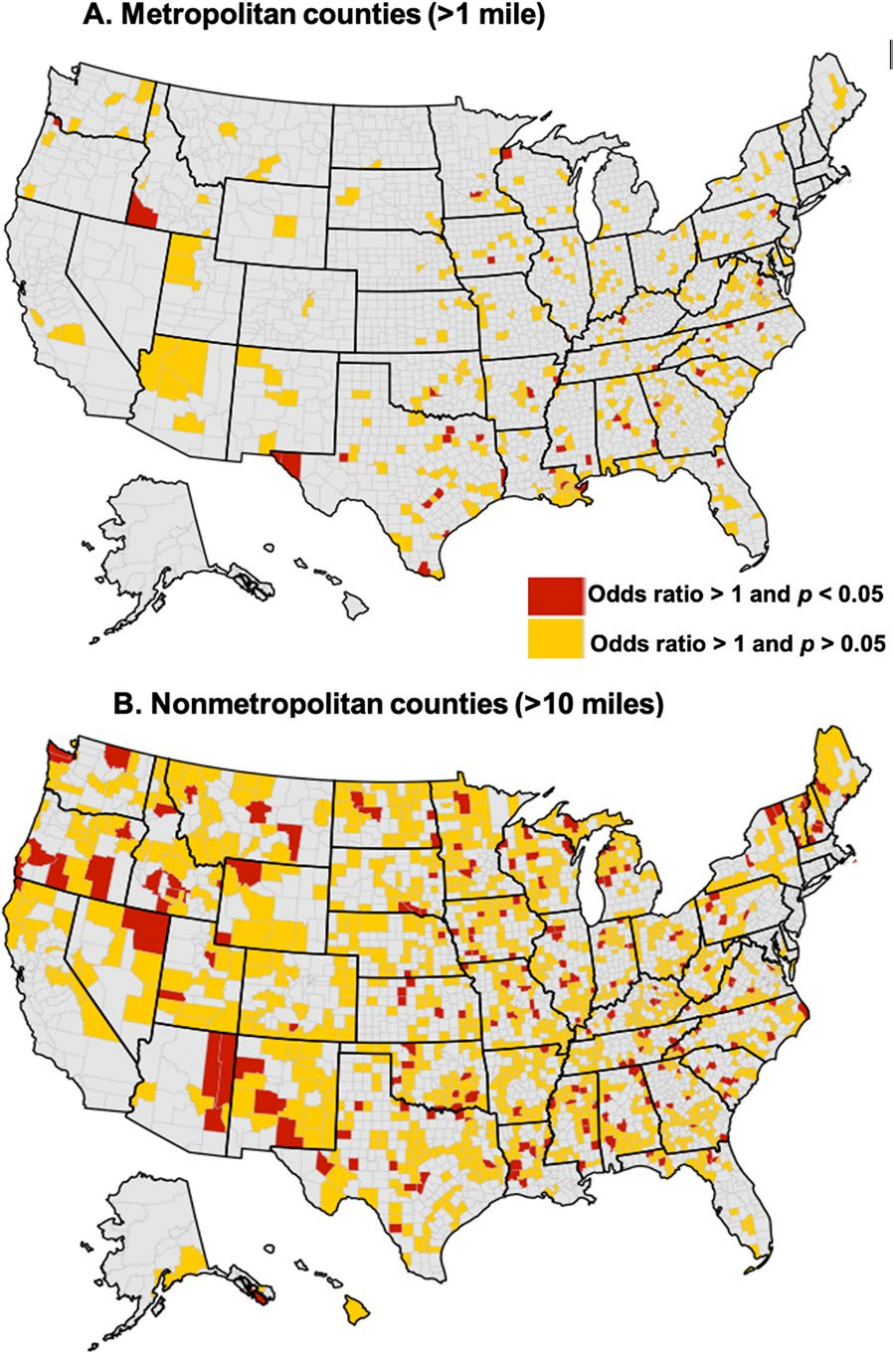
Metropolitan and nonmetropolitan Counties with Disparities in Access to Health Care Facilities. We calculated the odds of having a distance > 1 mile in metropolitan counties (**A**) or > 10 miles in nonmetropolitan counties (**B**) to the closest facility for low-income residents, compared to non-low-income residents. Low income was defined as household income < 200% Federal Poverty Level. Red indicates counties where disparities were significant at the p < 0.05 level and yellow indicate counties where disparities were non-significant at the p > 0.05 level. Grey indicates no disparity identified [7–11]

Among 1166 metropolitan counties, we identified 43 counties where low-income residents had significantly higher odds of having a driving distance > 1 mile to the closest facility. In 164 additional metropolitan counties, low-income residents had a higher likelihood of having a driving distance > 1 mile to the closest facility, though not statistically significant. (Fig. 1A) The forementioned 207 metropolitan counties accounted for almost 27 million Americans. Table 1 lists the most populated metropolitan counties with significant disparities in access to health care facilities, including 6 counties in Texas.

**Table 1:** Metropolitan Counties with Significant Disparities in Access to Health Care Facilities at the 1 Mile Threshold

County Name	State	Population	Proportion of non-low-income population with distance > 1 mi (%)	Proportion of low-income population with distance > 1 mi (%)	Odds ratio of distance > 1 mi for low-income vs. non-low-income
Dallas	Texas	2,635,516	39	44	1.18 (1.12, 1.14)
Collin	Texas	1,034,730	37	42	1.21 (1.07, 1.24)
San Francisco	California	881,549	2	4	1.93 (1.43, 1.94)
Hidalgo	Texas	868,707	60	67	1.35 (1.22, 1.34)
El Paso	Texas	839,238	42	45	1.11 (1.02, 1.14)
Richmond	Virginia	230,436	26	31	1.32 (1.08, 1.34)
Clay	Florida	219,252	73	80	1.48 (1.14, 1.44)
Monroe	Pennsylvania	170,271	81	86	1.41 (1.05, 1.44)
Guadalupe	Texas	166,847	68	76	1.50 (1.12, 1.54)
Ector	Texas	166,223	51	57	1.27 (1.02, 1.24)
Coweta	Gorgia	148,509	76	83	1.49 (1.08, 1.44)
Hardin	Kentucky	110,958	60	67	1.39 (1.05, 1.34)

The table lists counties categorized as metropolitan by the National Center for Health Statistics Urban–Rural Classification Scheme for Counties, with a population of at least 10,000 people, and where low-income residents had a significantly higher risk of having a driving distance > 1 mile to the nearest health care facility, compared to non-low-income residents. Low income was defined as household income < 200% Federal Poverty Level. Counties were ranked by decreasing population

Among 1967 nonmetropolitan counties, we identified 205 counties where low-income residents had significantly higher odds of having a driving distance > 10 miles to the closest facility. In 542 additional nonmetropolitan counties, low-income residents had a higher likelihood of having a driving distance > 10 miles to the closest facility, though not statistically significant. (Fig. 1B) These forementioned 747 nonmetropolitan counties accounted for almost 20 million Americans.

## Discussion

Our study has characterized income disparities in driving distance to health care facilities across the entire US. Our analyses demonstrate that in 954 counties (207 metropolitan counties and 747 nonmetropolitan counties) representing over 14% of the US population, low-income residents have poorer access to health care facilities. Our analyses demonstrate the high prevalence of structural disparities in health care access across the entire US, which contribute to the perpetuation of disparities in health care outcomes [1]. Although not the only type of obstacles to health care access, geographic barriers are particularly relevant for low-income populations because they are less likely to own a car and more likely to reside in areas with lower access to public transportation [6]. Investing in equitable health care access in the US should be a leading priority, particularly in the COVID-19 era.

## Conclusion

Our analyses demonstrate the high prevalence of structural disparities in health care access across the entire US, which contribute to the perpetuation of disparities in health care outcomes.

## Limitation

Non-significant disparities are presented because our 1% sampling of the US population may have resulted in under-power to detect disparities among nonmetropolitan counties at the statistical significance level.

## Data Availability

The datasets used and/or analyzed during the current study are available from the corresponding author on reasonable request.

## Abbreviation

COVID-19: Coronavirus disease 2019
